# The Effects of Yeast Protein on Gut Microbiota in Mice When Compared with Soybean Protein and Whey Protein Isolates

**DOI:** 10.3390/nu16030458

**Published:** 2024-02-05

**Authors:** Xuewei Zhou, Li Liang, Baoguo Sun, Ku Li, Hui Guo, Yuyu Zhang

**Affiliations:** 1 Key Laboratory of Geriatric Nutrition and Health, Beijing Technology and Business University, Ministry of Education, Beijing 100048, China; 20220100@btbu.edu.cn (X.Z.); gcfll@126.com (L.L.); sunbg@btbu.edu.cn (B.S.); 2 Food Laboratory of Zhongyuan, Beijing Technology and Business University, Beijing 100048, China; 3 Key Laboratory of Flavor Science of China General Chamber of Commerce, Beijing Technology and Business University, Beijing 100048, China; 4 National Key Laboratory of Agricultural Microbiology Core Facility, Angel Yeast Co., Ltd., Yichang 443003, China; liku@angelyeast.com (K.L.); guohui@angelyeast.com (H.G.)

**Keywords:** yeast protein, gut microbiota, *Parabacteroides*, immunity, inflammation

## Abstract

Different protein sources can impact gut microbiota composition and abundance, and also participate in health regulation. In this study, mice were gavaged with yeast protein (YP), soybean protein isolate (SPI), and whey protein isolate (WPI) for 28 days. Body weights showed similar patterns across different protein administration groups. The ileum in YP-supplemented mice exhibited good morphology, and tight-junction (TJ) proteins were slightly upregulated. Immunoglobulin (Ig)A, IgM, and IgG levels in the ileum of different protein groups were significantly increased (*p* < 0.05). Interleukin (IL)-10 levels were significantly increased, whereas IL-6 levels were significantly reduced in the YP group when compared with the control (C) (*p* < 0.05). Glutathione peroxidase (GSH-Px) levels in the ileum were significantly increased in the YP group (*p* < 0.05). These results indicate that YP potentially improved intestinal immunity and inflammatory profiles. The relative abundances of *Parabacteroides*, *Prevotella*, and *Pseudobutyrivibrio* in the YP group were more enriched when compared with the C and SPI groups, and *Parabacteroides* was significantly upregulated when compared with the WPI group (*p* < 0.05). Overall, the results indicate that YP upregulates the beneficial bacteria and improves ileal immunity and anti-inflammatory capabilities.

## 1. Introduction

Dietary protein affects the composition and function of different intestinal microorganisms, while gut microbes are highly correlated with health [1,2]. Dietary proteins such as casein, soybean, chicken, pork, beef, fish, egg, and whey exert different effects on gut microbiota structures and abundance, e.g., colonic microbiota in rats fed soy protein and casein showed similar microbiota composition [3]. Chao index in the colonic microbiota of mice fed soybean protein was significantly decreased when compared with animals fed casein [4]. Moreover, gut microbiota regulation by protein sources was independent of dietary fat and carbohydrate effects [5]. These findings suggest that protein sources have an important impact on the gut microbiota. Additionally, different protein sources also impacted on liver triglyceride (TG) levels, which decreased in rats fed grass carp and chicken, while the levels increased in rats fed pork and beef [6]. One key function of the gut microbiota is to generate short-chain fatty acids (SCFAs) via fermentation, especially in the colon. SCFAs exert considerable beneficial effects such as improving metabolic function, ameliorating immune dysfunction, and inhibiting insulin resistance [7]. Dietary protein sources can also affect nitrogen metabolism, and antioxidant and immune functions, changes which are related to the gut microbiota [8]. The Bacteroidetes phylum appears to help regulate the immune system via the intestines as its colonization was related to Th1/Th2 immune responses [9]. Weaned piglets fed enzyme-treated soybean meal had increased serum superoxide dismutase (SOD) levels and decreased interleukin (IL)-1β levels when compared with animals administered hydrolyzed wheat protein [8]. However, no differences in immunoglobulin (Ig)A, IgG, or IgM levels were identified between fermented soybean, hydrolyzed wheat protein, and enzyme-treated soybean meal. Different dietary protein sources can also affect tight junction (TJ) proteins in intestinal tissues; a previous study showed that occludin expression in the colon tissues of mice fed soybean protein and a casein diet was higher when compared with a whey-protein concentrate diet group, and total SCFAs were not significantly different across groups [10]. These observations suggest that different dietary proteins may affect the gut microbiota, and correlate with immunity and inflammation indices in intestinal tissues.

Yeast protein (YP) is extracted from the yeast Saccharomyces cerevisiae, the protein content of which can be as high as 70%, and the essential amino acid scores were close to the FAO/WHO ideal model [11,12]. Furthermore, as protein comes from single cells, YP has shorter production cycles, production processes are energy efficient, and production does not require arable land resources [13]. In an in vitro trial, YP concentrate had a mean true ileal indispensable amino acid digestibility of 85.8%, which was higher when compared with corn and pea protein [14]. In an in vivo trial, muscle fiber cross-sectional areas in the quadriceps femoris tissue of aging mice were increased after YP supplementation, and the α-diversity of the intestinal microbiota was increased [15]. These observations indicate that improvements in muscle aging by YP supplementation is related to gut microbiota alterations. In other research, using 40% YP to replace soybean protein when producing meat analogs, the brightness, whiteness, hardness, and chewiness had improved when compared with 100% soybean protein meat analogs [11]. However, the effects of YP on the gut microbiota and its relationship with intestinal health that was induced by gut microbiota alterations remain unclear. Therefore, examining the regulatory effects of YP on gut microbiota composition and function is required to understand its impact on nutritional function.

To this end, we investigated the effects of YP on gut microbiota composition and function in mice and compared the effects to those of soybean protein isolate (SPI) and whey protein isolate (WPI). We also examined YP regulatory effects and mechanisms on intestinal immunoglobulin, inflammatory, and oxidative stress levels in the mice. The observations potentially highlight the benefits of YP as a dietary protein source.

## 2. Materials and Methods

### 2.1. Experiment Design

Five-week-old C57BL/6J mice (male, *n* = 48) used in animal studies were purchased from Beijing Vital River Laboratory Animal Technology (Beijing, China). Animals had ad libitum access to feed and water, and were housed four mice per individually ventilated cage at 20–26 °C and 40–70% humidity in 12 h/12 h light/dark cycles. A standard commercial chow (Beijing Keao Xieli Feed Co., Ltd., Beijing, China) comprising 24.02% protein, 12.95% fat, 63.03% carbohydrate, and 3.44 kcal/g energy was used to feed mice. Animals were acclimated under rearing conditions for 7 days before studies started, and were then randomly divided into Control (C), YP, SPI, and WPI groups of 12 mice/group. YP, SPI, and WPI powders were purchased from AngelYeast Co., Ltd. (Yichang, China), Shansong Biological Products Co., Ltd. (Linyi, China), and Fonterra Co-operative Group Limited (New Zealand), respectively. During studies, the mice received 200 μL (0.1 g/mL) [15] of the respective protein diet by gavage every day in the morning. The mice in group C received 200 μL of distilled water. Body weights were recorded once per week. The water used for drink and gavage was autoclaved in advance.

Studies followed the relevant guidelines and regulations of the Institutional Animal Care and Use Committee of Beijing Vital River Laboratory Animal Technology Co., Ltd. (No. P2022039).

### 2.2. Sample Collection

Fecal samples were collected on day 28 and immediately stored at −80 °C for further analysis. Then, the mice were fasting for 6 h. Animal sacrifices were humanely performed using carbon dioxide. Intestinal tissues were then immediately removed, and the ileum was identified and separated. After rinsing in sterile phosphate buffered saline (PBS) at 4 °C, one sample was rapidly frozen in liquid nitrogen and stored at −80 °C, while a 0.5 cm sample was fixed in 4% paraformaldehyde (Solarbio, Beijing, China) for histology.

### 2.3. Histopathology

Intestinal epithelial morphology was used to assess the physical barrier in the gut and its impact on nutrient absorption. Fixed ileum tissue was embedded in paraffin blocks, after which 5 μm sections were dewaxed in xylene, absolute ethanol (Sinopharm Chemical Reagent Co., Ltd., Shanghai, China), and 75% ethanol. Sections were then stained in hematoxylin–eosin stain (H&E, Wuhan Servicebio Technology Co., Ltd., Wuhan, China) to observe epithelial morphology in ileum tissue. Slides were evaluated and photographed under a BL-180Z light microscope equipped with a microscope video camera controller (Beijing Century COSSIM Scientific Instrument CO., Ltd., Beijing, China). Villus length and crypt depth dimensions were measured using ImageJ software (NIH, Bethesda, MD, USA).

### 2.4. Enzyme-Linked Immunosorbent Assay (ELISA)

IgA, IgG, IgM, IL-6, IL-10, IL-4, and tumor necrosis factor-α (TNF-α) levels in ileum tissues were measured using mouse ELISA kits according to the manufacturer’s instructions. In addition, antioxidant enzymes, including glutathione peroxidase (GSH-Px), catalase (CAT), SOD, and lipid peroxidation product malondialdehyde (MDA) levels, which potentially influenced oxidative stress, were also measured using mouse ELISA kits. Ileum samples (10 mg) were cut into small pieces and homogenized in 100 μL PBS. TP levels were determined using bicinchoninic acid assay (BCA) protein quantification ELISA kits (Shanghai Enzyme-linked Biotechnology Co., Ltd., Shanghai, China). A microtiter plate reader (RT-6100, Rayto, Shenzhen, China) was used to measure optical density at 450 nm.

### 2.5. 16S rRNA Sequencing

Bacterial DNA from fecal samples was isolated using MOBIO PowerFecalTM DNA Isolation Kits (MOBIO Laboratories, Carlsbad, CA, USA) according to the manufacturer’s specifications. The DNA was amplified using a nested polymerase chain reaction (PCR) method. The V4 hypervariable region of the bacterial 16S rRNA gene was amplified using the following primers: 515F (5′-AYTGGGYDTAAAGNG-3′) and 806R (5′-TACNVGGGTATCTAATCC-3′). The PCR reaction system was performed using an Applied Biosystems 2720 Thermal Cycler following a previous study [16].

Amplicon DNA was purified using AMPure XP beads (Beckman Coulter Genomics, Danvers, MA, USA) and quantified using Qubit instrumentation (Invitrogen, Carlsbad, CA, USA). Paired-end sequencing was performed on a NovaSeq (Illumina, Inc., San Diego, CA, USA) and data were analyzed in QIIME2 (Version 2019.4). Raw sequence data were quality-filtered using Trimmomatic (Version 0.33), and primer regions were trimmed using the Cutadapt (Version 1.9.1) plugin in QIIME2. Then, the sequences were denoised, merged, and chimera removed using the DATA2 plugin. Non-singleton amplicon sequence variants (ASVs) were aligned with mafft and used to construct a phylogeny with fasttree2. The DATA2 algorithm was used to process and obtain specific sequences from sequences without primer regions. Valid data for samples were obtained from quality control filtering. High-quality sequences were then clustered using alpha (α)-diversity (Chao1 and Shannon indexes), beta (β)-diversity metrics (Bray–Curtis metrics), and the weighted UniFrac that estimated using the diversity plugin with samples were rarefied to 3806. The Kruskal–Wallis test and permutational multivariate analysis of variance (PERMANOVA) were used for testing the significance of α-diversity and β-diversity.

### 2.6. SCFAs Measurements in Feces

Six SCFAs (acetic acid, propionic acid, butyric acid, isobutyric acid, valeric acid, and isovaleric acid) were measured using gas chromatography–mass spectrometry (GC–MS, Thermo Fisher Scientific, Waltham, MA, USA) according to a previous method, with some modifications [17,18]. Fecal samples (50 mg) were dissolved in 15% phosphoric acid (50 μL) (Sinopharm Chemical Reagent Co., Ltd. Shanghai, China) and then mixed with 100 μL (125 μg/mL) of the internal standard 4-methylvaleric acid (Sigma-Aldrich, Darmstadt, Germany) and 400 μL of ether (Greagent, Shanghai, China). Samples were homogenized for 1 min, centrifuged for 10 min (4 °C and 12,000 rpm), and supernatants transferred to vials for GC–MS analyses. Absolute SCFA levels were determined using calibration curves for each analyte. Trace 1300 GC instrumentation, equipped with ISQ 7000 MS (Thermo Fisher Scientific, MA, USA) was used for SCFA analysis.

### 2.7. Western Blot

TJ expression in ileum tissues was used to evaluate gut barrier function in different protein-supplemented groups. Zonula occludens-1 (ZO-1), mucin-2, and occludin levels were measured by Western blotting. TP was extracted from ileum tissues in 150 μL of radioimmunoprecipitation assay buffer (SinoGene Scientific Co., Ltd., Beijing, China) and quantified using a BCA protein quantification kit (SinoGene Scientific Co., Ltd., Beijing, China). Quantified protein (30 μg) was then separated using sodium dodecyl sulphate-polyacrylamide gel electrophoresis and transferred to polyvinylidene difluoride membranes (0.22 μm, BioTrace, New York, NY, USA). Antibodies against occludin (ab216327) and MUC-2 (ab272692) were purchased from Abcam (Cambridge, UK). The ZO-1 (21773-1-AP) antibody was purchased from Proteintech (Wuhan, China). After blocking in fast protein-free block buffer (SinoGene), the membranes were incubated with the respective antibodies at room temperature for 1.5 h, washed, and respective secondary antibodies (AS063, AS0664, Wuhan, China) added for 1 h at room temperature. Enhanced chemiluminescent solution (29050, Engreen, Beijing, China) was then added to the membranes in the dark. β-Actin (1:3000, AC028, Abclonal, Wuhan, China) was used as an internal reference. Protein band densitometry was performed using ImageJ software (NIH, Bethesda, MD, USA).

### 2.8. Statistical Analyses

Data were compared using one-way analysis of variance (ANOVA) in SPSS 17.0 (IBM, Chicago, IL, USA), with differences between groups analyzed using Tukey multiple comparisons tests. Heatmap, Principal Coordinates Analysis (PCoA), and Spearman correlation were analyzed on microbiome data, conducted in RStudio (Version 3.5.2, Boston, MA, USA). Figures were generated in GraphPad Prism (Version 9.0.0, GraphPad Software, San Diego, CA, USA).

## 3. Results

### 3.1. The Effects of Protein Supplementation on Mouse Body Weight

**Figure 1:**
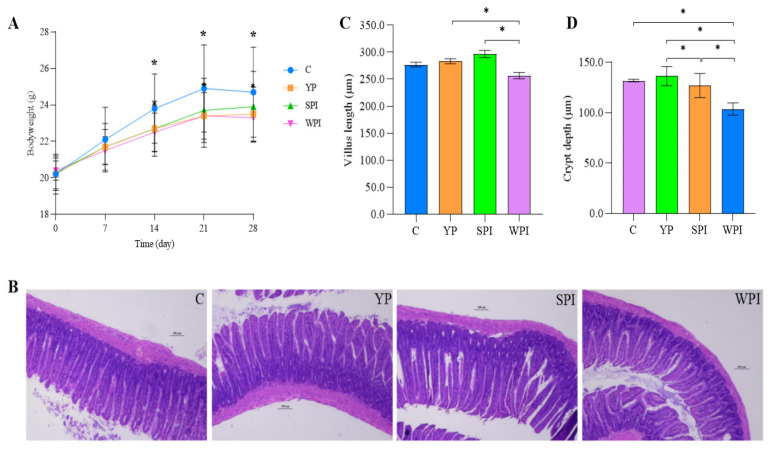
The effect of protein on body weight and the ileal morphology of mice. (**A**) Protein supplementation reduces the body-weight-increasing trends of mice (*n* = 12); (**B**) Hematoxylin–eosin staining of ileum (scale bar, 100 μm, *n* = 4); (**C**) Villus length of ileum tissues; (**D**) Crypt depth of ileum; The error bar in image A is the standard deviation, while in image (**C**,**D**) they represent the standard error of mean. C, control group; YP, yeast protein; SPI, soybean protein isolate; WPI, whey protein isolate. *p* values are calculated using the one-way ANOVA with Tukey’s post hoc test. *, *p* < 0.05.

Mouse body weights are shown in Figure 1A. An increasing trend was observed from 0 to 21 days in all groups, whereas no changes were recorded on day 28. After gavage with protein, the body weight in protein-supplemented mice was lower than that in group C. Body weights in control mice were significantly higher (*p* < 0.05) when compared with protein-supplemented groups from day 14, and subsequently, no significant differences were identified between protein-supplemented groups.

### 3.2. Histological Observations

To evaluate intestinal epithelial integrity, H&E staining was used to analyze intestinal morphology in ileum tissues (Figure 1B), with villus length and crypt depth measured (Figure 1C,D). Villus length in YP and SPI groups was significantly longer when compared with the WPI group (*p* < 0.05), but villus density in YP, SPI, and WPI groups was not significantly changed when compared with group C. Crypt depth in YP-supplemented mice was significantly higher when compared with SPI and WPI groups (*p* < 0.05), while no differences were observed when compared with group C. WPI-supplemented mice had shorter villi and shallower crypts in their ileal intestinal epithelia.

### 3.3. Immune, Inflammation, and Oxidative Stress Levels in Ileum Tissues

**Figure 2:**
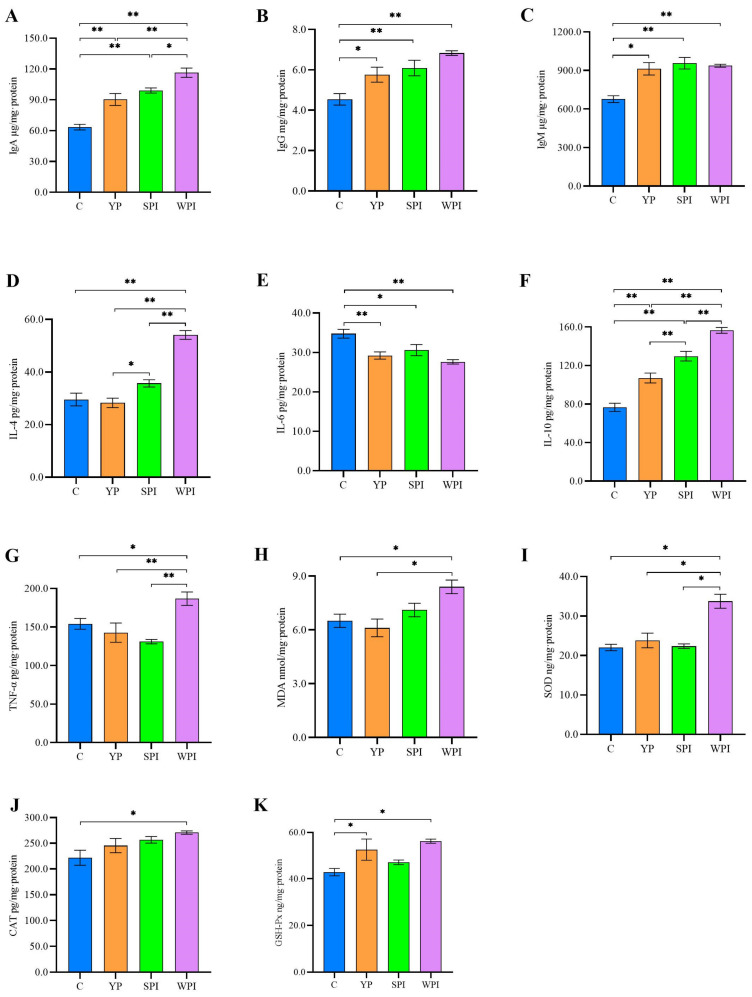
Average levels of immunoglobulin, inflammation, and oxidative stress in ileum tissues of C57BL/6J mice. (**A**) immunoglobulin A; (**B**) immunoglobulin G; (**C**) immunoglobulin M; (**D**) interleukin-4; (**E**) interleukin-6; (**F**) interleukin-10; (**G**) tumor necrosis factor (TNF)-α; (**H**) malondialdehyde; (**I**) superoxide dismutase; (**J**) catalase; (**K**) glutathione peroxidase. *n* = 3. C, control; YP, yeast protein; SPI, soybean protein isolate; WPI, whey protein isolate. *p* values are calculated using the one-way ANOVA with Tukey’s post hoc test. *, *p* < 0.05; **, *p* < 0.01. Error bars represent the standard error of mean.

Evaluating interleukin and immunoglobulin variations provided a deeper understanding of inflammatory cytokines and immune responses in mice gavaged with YP, SPI, and WPI. Immunoglobulin and cytokine levels in ileal tissues were measured using ELISA (Figure 2). Ileal IgA, IgG, and IgM levels in YP-supplemented mice were significantly increased when compared with group C. IgG and IgM levels in SPI and WPI-supplemented mice also showed significant increases when compared with group C (*p* < 0.05), but no differences when compared with group YP. Only IgA levels in WPI-supplemented mice were significantly higher than in group YP (*p* < 0.05) (Figure 2A–C). For cytokines, IL-4 and IL-10 levels in ileum tissues from YP mice were significantly lower than in the SPI and WPI groups (*p* < 0.05); however, IL-10 levels were higher when compared with group C (Figure 2D–F). IL-6 levels in YP, SPI, and WPI groups were significantly reduced (*p* < 0.05) when compared with group C, but no differences were recorded between protein-supplemented groups. TNF-α levels in the WPI group were significantly higher when compared with other groups (*p* < 0.05), but no differences were recorded among C, YP, and SPI groups (Figure 2G).

Antioxidant levels in ileum tissues were examined using ELISA (Figure 2H–K). MDA and SOD levels in the YP group were significantly lower when compared with the WPI group (*p* < 0.05), and no differences were recorded between YP and SPI groups in terms of SOD, GSH-Px, CAT, and MDA levels. Additionally, GSH-Px levels in the YP group were significantly higher when compared with group C (*p* < 0.05). The levels of three antioxidants and MDA in WPI mice were significantly higher than in group C mice (*p* < 0.05).

### 3.4. Gut Microbiota Composition and Diversity

**Figure 3:**
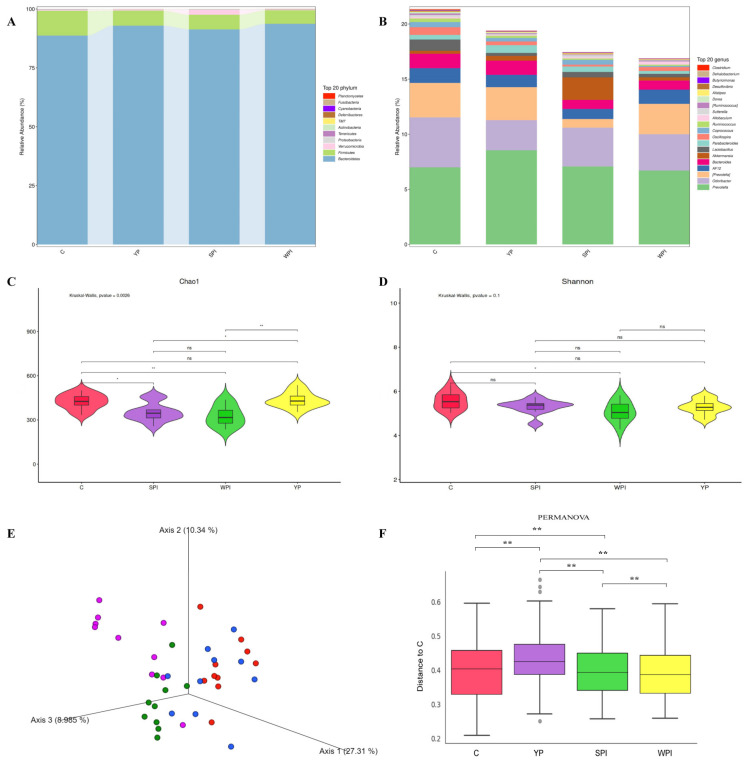
The abundance and diversity of microbiome in fecal samples. (**A**) The relative abundance of the top 20 microbes at phylum level; (**B**) The relative abundance of top 20 microbes at genus level; (**C**) Chao1 diversity index of gut microbiota in mice of C, YP, SPI, and WPI groups; (**D**) Shannon diversity index of gut microbiota in mice of C, YP, SPI, and WPI groups; (**E**) Bray–Curtis PCoA of gut microbiota in C, YP, SPI, and WPI gavage mice; (**F**) Bray–Curtis distance of gut microbiota between the three protein groups and the control group. *n* = 10. C, control; YP, yeast protein; SPI, soybean protein isolate; WPI, whey protein isolate. Pairwise *p* values are calculated using the nonparametric Kruskal–Wallis test with Tukey’s post-hoc test. PCoA, principal coordinate analysis; PERMANOVA, permutational multivariate analysis of variance. ns, not significant; *, *p* < 0.05; **, *p* < 0.01.

Fecal microbiota abundance and diversity levels are shown in Figure 3. Bacteroidetes were the most abundant phyla in all groups, followed by Firmicutes and Verrucomicrobia (Figure 3A). YP, SPI, and WPI groups had higher relative abundance of Bacteroidetes and lower Firmicutes than group C at the phylum level. Higher relative abundance of Verrucomicrobia was observed in the SPI group than in the C, YP, and WPI groups. In total, 91 genera were identified from the fecal samples; the relative abundances of the top 20 genera are shown in Figure 3B. A higher relative abundance of *Prevotella* was observed in the YP group when compared with the C, SPI, and WPI groups, but with lower *Odoribacter* levels. The relative abundance of *Parabacteroides* in the YP group was increased and higher than in the C, SPI, and WPI groups. In the SPI group, the relative abundance of *Akkermansia* genus was higher than in the C, YP, and WPI groups. The bacterial richness and diversity were evaluated and are shown in Figure 3C–E. For α-diversity, the Chao1 index was not significantly different between the C and WPI groups (Figure 3C). Both YP and SPI supplements significantly decreased the Chao1 index when compared with C and WPI groups (*p* < 0.05) (Figure 3C), which indicated a decreasing richness of microbial community abundance after YP and SPI supplementation. However, the Shannon index in the SPI group was significantly lower than in group C, whereas no significant differences were identified between the C, YP, and WPI groups (Figure 3D), which indicated no changes in microbial community diversity among the C, YP, and WPI groups. For β-diversity, PCoA, based on Bray–Curtis distances related to β-diversity, indicated that overall bacterial structures in the C and WPI groups had clustered together, whereas they had separated in the YP and SPI groups (Figure 3E). Furthermore, PERMANOVA results calculated from Bray–Curtis distances indicated significant differences among protein-supplemented and C groups (Figure 3F).

### 3.5. Gut Microbiota Structure and Abundance

**Figure 4:**
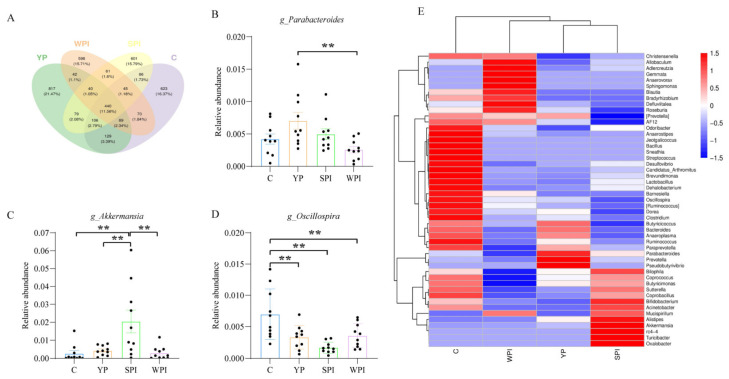
The Venn and relative abundance of each taxonomic genus in different dietary proteins. (**A**) The unique OTUs among groups; (**B**–**D**) Representative significantly changed gut microbes at genus levels; (**E**) Heatmap of the top 50 abundant genera in the gut microbiota. *n* = 10. C, control; YP, yeast protein; SPI, soybean protein isolate; WPI, whey protein isolate. **, *p* < 0.01.

Venn diagram analyses indicated 623, 817, 601, and 598 unique operational taxonomic units (OTUs) in C, YP, SPI, and WPI groups, respectively. Also, 440 OTUs were shared between groups (Figure 4A). We also identified the top 50 abundant genera from each group (Figure 4E); most of the genera in protein-supplemented groups were reduced when compared with group C. Relative abundance of genera *Parabacteroides*, *Prevotella*, and *Pseudobutyrivibrio* was enriched in the YP group (Figure 4E), but *Parabacteroides* levels were significantly higher than in the WPI group (*p* < 0.05) (Figure 4B). The relative abundance of genera *Christensenella*, *Odoribacter*, and *Mucispirillum* was reduced when compared with SPI and WPI groups, but no significant differences were observed. In addition, the relative abundance of genus Akkermansia in the SPI group was significantly upregulated when compared with the other groups (Figure 4C). The relative abundance of genera *Oscillospira* in protein-supplemented groups was significantly decreased when compared with group C (Figure 4D).

### 3.6. ZO-1, Occludin, and Mucin-2 Expression Levels in Ileum Tissues

**Figure 5:**
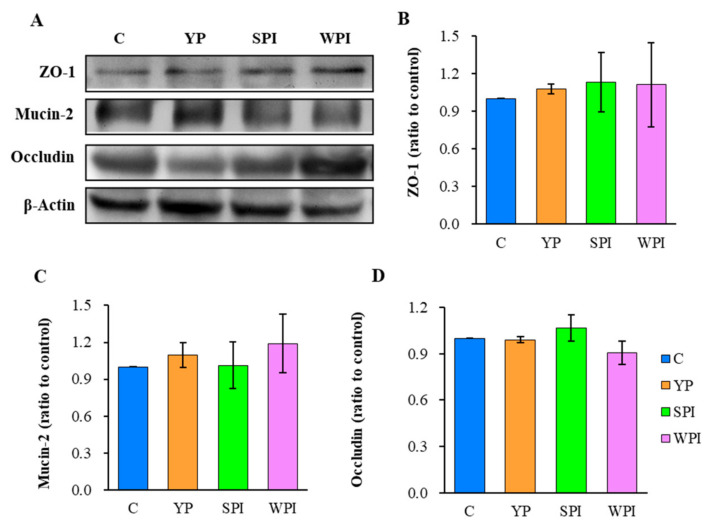
Expression levels of gut barrier-associated proteins ZO-1, mucin-2, occludin in ileum tissues of C, YP, SPI, and WPI groups using Western blot with quantitative analysis. (**A**) immunoblots and quantification; (**B**) ZO-1, zonula occludens-1; (**C**) Mucin-2; (**D**) Occludin. *n* = 3; C, control; YP, yeast protein; SPI, soybean protein isolate; WPI, whey protein isolate. Error bars represent the standard error of mean.

To evaluate barrier function and permeability, ZO-1 and occludin expression levels in ileum tissues were measured (Figure 5). Mucin-2, which functions in mucus layers, was also measured to assess the epithelium barrier [19]. ZO-1 and mucin-2 expression levels in the YP group were slightly increased when compared with group C, and were similar to WPI group levels. However, no significant differences were recorded across groups.

### 3.7. SCFA Levels in Feces

**Figure 6:**
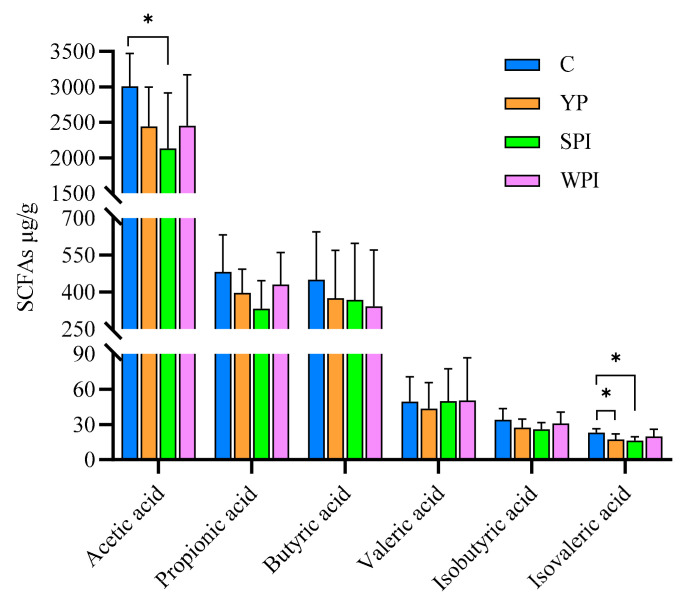
The contents of SCFAs in fecal samples. For C and YP group, *n* = 10; For SPI and WPI group, *n* = 9. C: control; YP: yeast protein; SPI: soybean protein isolate; WPI: whey protein isolate. *p* values are calculated using the one-way ANOVA with Tukey’s post hoc test. *, *p* < 0.05.

SCFA levels in feces are shown in Figure 6. Acetic acid had the highest levels, followed by propionic acid and butyric acid, which were similar. Propionic acid, butyric acid, isobutyric acid, and valeric acid levels showed no significant differences across protein-supplemented groups. Acetic acid and isovaleric acid levels showed no significant differences among YP, SPI, and WPI groups, while acetic acid levels in the SPI group were significantly lower when compared with group C (*p* < 0.05), and isovaleric acid levels in the YP and SPI groups were significantly decreased when compared with group C.

### 3.8. Correlations Analysis

**Figure 7:**
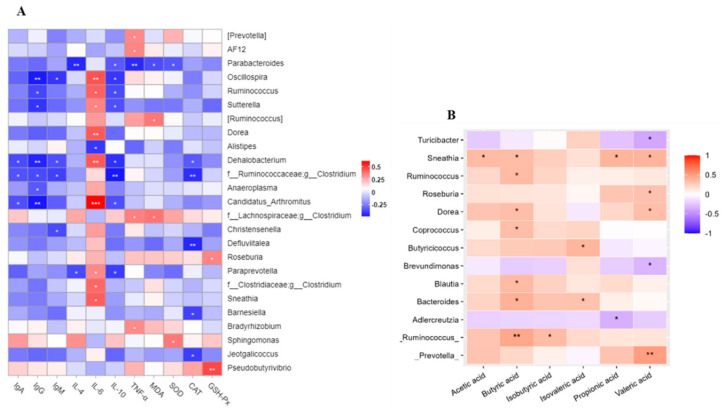
Significant correlations analysis. (**A**) Significant correlations among genera with cytokine, immunoglobulin, and antioxidant. (**B**) Significant correlations between genera and SCFAs. *n* = 10. IgA, immunoglobulin A; IgG, immunoglobulin G; IgM, immunoglobulin M; IL-4, interleukin-4; IL-6, interleukin-6; IL-10, interleukin-10; TNF-α, tumor necrosis factor -α; MDA, malondialdehyde; SOD, superoxide dismutase; CAT, catalase; GSH-Px, glutathione peroxidase. *, *p* < 0.05; **, *p* < 0.01, ***, *p* < 0.001. Without unidentified and unclassified OUTs.

A correlation heatmap (performed using Spearman’s correlation analysis) identified relationships between the gut microbiota, cytokine, immunoglobulin, antioxidant, and MDA levels (Figure 7A). A decreased abundance in genera was negatively correlated with immunoglobulin levels (*p* < 0.05). IL-6 levels were negatively correlated with the abundance of the genus Alistipes, whereas they were significantly and positively related to nine other genera (*p* < 0.05). However, IL-10 levels showed significantly negative correlations with eight genera, with six genera positively related to IL-6. TNF-α levels showed significantly negative correlations with genus Parabacteroides (*p* < 0.01). Increased abundance of Roseburia and Pseudobutyrivi was positively correlated with GSH-Px levels (*p* < 0.05). CAT levels showed negative correlations with five genera, whose relative abundances were not significantly altered.

Correlations between specific taxa and SCFAs are shown in Figure 7B. Valeric acid levels were significantly correlated with the enriched genera, *Sneathia*, *Roseburia*, *Dorea*, and *Prevotella* (*p* < 0.05), while *Dorea* and *Sneathia* were also positively correlated with butyric acid levels (*p* < 0.05). Additionally, butyric acid levels were positively correlated with *Ruminococcus*, *Coprococcus*, *Blautia*, and *Bacteroides* (*p* < 0.05). A decreasing abundance in *Turicibacter* and *Brevundimonas* taxa was negatively correlated with valeric acid levels, and *Adlercreutzia* was negatively correlated with propionic acid levels (*p* < 0.05).

## 4. Discussion

### 4.1. YP, SPI, and WPI Supplementation Had Similar Effects with Respect to Body Weight Gain and Epithelial Barrier Function

Protein supplementation generated less body weight gain in mice, in agreement with a previous study showing that protein induced higher satiation levels when compared with carbohydrates [20]. A lower body weight gain was also observed in mice supplemented with soybean protein [21]. In our study, all mouse groups had ad libitum access to water and feed, and were gavaged with the same proportion of YP, SPI, and WPI, whereas the control group was gavaged with water, which may have affected dietary intake and generated lower body weight gains. Unfortunately, the feed intake of mice was not recorded; this should be examined in future studies.

ZO-1 helps maintain epithelial integrity, whereas mucin-2 enhances gut homoeostasis [22], and protein supplementation, for example, soybean protein, can increase occludin expression [23]. In this study, YP, SPI, and WPI animals showed similar or little increases in ZO-1 and mucin-2 expression trends when compared with group C. YP and SPI mice showed a similar occludin trend, and a decreasing trend was observed in the WPI group. Accordingly, the villus length was significantly longer in the YP-supplemented group than in the WPI group (*p* < 0.05), whereas crypt depth was significantly higher than in the SPI and WPI groups (*p* < 0.05); however, there was no difference between the YP and control groups. The increased villus length expands the surface area of the intestine and increases nutrient absorption, and the deeper crypts have higher secretion capacity [24,25]. Therefore, YP could promote intestinal absorption of nutrients, compared with SPI and WPI.

### 4.2. Gut Microbiota Alterations and SCFAs’ Generation

Protein consumption in diets can drive microbial fermentation processes since it affects how much protein reaches the colon and participates in fermentation [1]. Additionally, dietary protein sources may impact protein fermentation in the colon due to protein digestibility [26]. Bacteroidetes and Firmicutes are the primary phyla in the intestinal microbiome. In our study, protein (YP, SPI, and WPI) supplementation increased the relative abundance of Bacteroidetes phyla. The relative abundance of *Prevotella* and Bacteroidetes was increased after YP supplementation; however, *Prevotella* was reportedly linked to a vegetable-rich diet [27]. The succinate pathway that is present in the abundant phylum Bacteroidetes is the most abundant route to generate propionate [28]. The Chao1 index in the SPI and YP groups were significantly lower than in the C and WPI groups. A previous study reported that feeding mice with soybean protein reduced this index when compared with casein and processed pork protein [2]. In terms of β-diversity distances, we observed that the YP group was similar to the SPI group, and was separated from control and WPI groups. Therefore, YP may impact the gut microbiota by upregulating probiotic abundance (i.e., *Prevotella* and *Parabacteroides*). Our Venn community analysis indicated that common OTU numbers were less than the unique OTUs in each group. Although *Parabacteroides*, *Prevotella*, *and Pseudobutyrivibrio* genera in the YP group were more enriched when compared with SPI and WPI groups, only relevant *Parabacteroides* abundance in the YP group was significantly higher (*p* < 0.05). A previous study reported that supplementation with casein, milk protein, and higher protein-portion feeding increased *Parabacteroides* genera [5]. Thus, YP supplementation to mice diets generated similar phylum composition and increased beneficial bacteria at the genus level.

### 4.3. YP Increases Immune and Antioxidant Status by Reducing Inflammation

In our study, immunoglobulins were significantly increased in the ileum of mice supplemented with YP, SPI, and WPI when compared with group C. A high-protein (casein) diet fed to C57BL/6 mice promoted high lamina propria IgA production when compared with high-fat and high-carbohydrate feeding, and increased IgA levels were dependent on T-cell-independent mechanisms and a modulated lamina propria-cytokine environment [29]. IL-10 is considered an anti-inflammatory cytokine, whose levels, derived from the intestinal epithelium, help maintain intestinal homeostasis due to IL-10′s positive regulation of the canonical nuclear factor-κB pathway [30]. Parabacteroides enhances and maintains Treg cells which produce IL-10, thus associating the cytokine with T-cell differentiation [31]. In our study, YP supplementation upregulated the relative abundance of Parabacteroides which may have generated higher IL-10 expression. Moreover, IL-4 expression in ileum tissues was not different when compared with the control groups, but was significantly lower than in the SPI and WPI groups. A comparison study showed that weanling C57BL/6 mice feeding with SPI as dietary protein downregulated the levels of IL-4 in the ileum when compared with a casein diet [32]. A previous study indicated that IL-4 acted as a pro-inflammatory cytokine when existing alone in normal mouse guts [33]. Compared to the control group, the levels of IL-4 in the YP group did not change, but were lower than in the SPI- and WPI-supplemented mice. But the anti-inflammatory cytokine IL-10 in the YP group was significantly higher than the control group, and also significantly lower than the SPI and WPI groups. Normally, the level of IL-6 in the intestine could maintain the immune response to a proper extent, and participate in the cross-talk regulation between the intestinal epithelium and the gut microbiota [34]. The probiotic treatment could reduce the level of IL- 6 in intestinal diseases [35]. In our study, the relative abundance of some probiotics was upregulated in protein-supplemented groups, such as *Parabacteroides*, *Akkermansia*, and *Roseburia*. Thus, our data suggested that YP potentially reduced inflammation in the ileum. YP supplementation showed no differences in antioxidant activity when compared with the SPI group. In a previous study, in a 12% soybean protein diet, SOD and CAT levels in duodenum and jejunum tissues were significantly decreased when compared with a control diet [36]. In addition, decreasing MDA (lipid peroxidation product) levels were observed in the YP-supplemented group, suggesting that YP improved antioxidant capacity in the ileum.

## 5. Conclusions

The results demonstrated that YP had a beneficial impact on gut microbiota composition and abundance, especially at genus levels. The relative abundance of Parabacteroides, which putatively generated SCFAs, was significantly upregulated in YP-supplemented mice. However, we recorded no differences in SCFAs across groups, which may have been related to the downregulation of SCFA-generating genus *Oscillospira* and low-dose protein supplementation. The YP supplement systemically reduced intestinal inflammation and enhanced immunity by decreasing IL-6 levels and increasing IL-10, IgA, IgG, and IgM levels in the ileum. Therefore, YP can be taken as a source of protein supplementation due to its beneficial effect on intestinal health.

## Data Availability

The data presented in this study are available from the corresponding author on reasonable request. The data are not publicly available due to [commercial restrictions].
